# The importance of material deprivation in type 2 diabetes incidence: longitudinal analyses in the Maastricht study

**DOI:** 10.1186/s12889-025-24263-1

**Published:** 2025-09-02

**Authors:** Rachelle Meisters, Annemarie Koster, Bengisu Sezer, Jeroen Albers, Marleen van Greevenbroek, Bastiaan de Galan, Hans Bosma

**Affiliations:** 1 Department of Social Medicine, Care and Public Health Research Institute (CAPHRI), Maastricht University, Duboisdomein 30, Maastricht, 6229GT the Netherlands; 2 Care and Public Health Research Institute (CAPHRI), Maastricht University, Maastricht, the Netherlands; 3 CARIM School for Cardiovascular Diseases, Maastricht University, Maastricht, the Netherlands; 4 Department of Internal Medicine, Maastricht University Medical Center+, Maastricht, the Netherlands; 5 Department of Internal Medicine, Radboud University Medical Center, Nijmegen, the Netherlands

**Keywords:** Material deprivation, Socioeconomic inequalities, Type 2 diabetes mellitus

## Abstract

**Aim:**

Type 2 diabetes mellitus (T2DM) is a common chronic disease that disproportionately affects groups with a low socioeconomic position (SEP). In addition to traditional SEP measures, material deprivation allows for further differentiation between people at the bottom of the socioeconomic hierarchy. This study therefore aimed to examine longitudinal associations between material deprivation and incident T2DM, independent of traditional SEP measures and childhood income inadequacy.

**Methods:**

Longitudinal data from 4,553 participants without T2DM at baseline in The Maastricht Study were used (52.4% female, mean age 57.9 (SD 8.6) years). Material deprivation was based on a 20-item questionnaire including lack of basic goods due to financial reasons, debts, economic strain and perceived financial problems. The highest completed educational level, household income equivalent, occupational position and childhood income inadequacy were measured at baseline by means of questionnaires. Incident T2DM was self-reported yearly (up to 12 years of follow-up). Associations were studied with Cox regression analyses.

**Results:**

Reported incident T2DM was 3.2% over 8.2 (median) years of follow-up. The risk for incident T2DM was significantly higher among people with material deprivation and for people with lower income, independent of education, occupation and childhood income inadequacy, with a hazard ratio (HR) of 1.38 (95%CI 0.90–2.10) and 1.71 (95%CI 1.08–2.70) for intermediate and high material deprivation, respectively.

**Conclusion:**

Material deprivation is a risk factor for incident T2DM, even after accounting for traditional SEP measures such as education, occupation and income. More attention is needed in research and practice for people facing material deprivation. Interventions aimed at reducing health inequalities should also target multiple SEP components and pathways.

**Supplementary Information:**

The online version contains supplementary material available at 10.1186/s12889-025-24263-1.

## Introduction

Type 2 diabetes mellitus (T2DM) has become a common chronic disease in all populations, and it disproportionally affects disadvantaged groups [1]. Socioeconomic inequalities have been systematically demonstrated in T2DM prevalence [2, 3] and incidence [4, 5]. A previous systematic review [4] showed a socioeconomic gradient with individuals with low socioeconomic position (SEP) facing increased risks for T2DM compared to individuals with high SEP. In explaining socioeconomic differences in T2DM prevalence and incidence, different indicators of adult SEP have been used [4]. Each indicator may influence T2DM prevalence and incidence through different pathways, with different strengths. In understanding and tackling the socioeconomic gradient in T2DM, multiple SEP indicators and their possible pathways should be analyzed and targeted [6].

Most studies that analyze the associations between SEP and T2DM incidence have used education, income and occupation or a combination hereof [4]. Previous research established that part of the health inequalities in T2D are explained by behavioral and metabolic risk factors for educational attainment and occupational class [6]. Furthermore, the theory of social causation stated that SEP affects health through a range of mechanisms for education, occupation and income. In explaining socioeconomic health inequalities, risk behavior and health awareness have been attributed to educational attainment. Occupational hazards and prestige status are linked to occupational class. Finally, affordability of health care, environmental hazards, and psychological burdens are related to income inequality [7].

A fourth indicator of adulthood SEP has also been associated with prevalent diabetes, namely material deprivation [3]. Material deprivation is a multifaceted concept, including lack of basic goods, debts, economic strain and perceived financial stress [3, 8–10]. This concept helps to move research beyond the effects of absolute income itself as it further specifies the effects of relative disadvantage (such as financial stress, a lack of material resources and basic resources, healthcare access and food insecurity) within a social context. In accordance with the material hypothesis, material deprivation in low SEP groups may have direct effects on health [9, 11]. Specifically, economic strain, or perceived income inadequacy, has been associated with poorer health independent of income levels [12]. More broadly, material deprivation has been associated with higher T2DM prevalence, independent of income, education or occupational level [3], but whether it impacts T2DM incidence is less well studied. Various studies have looked at material deprivation from an area level perspective [13–16]. One study included multiple SEP indicators based on individual respondents, but foremost considered wealth in terms of properties, assets, investments and savings [17]. Particularly because material deprivation further differentiates between people at the very bottom of the socioeconomic hierarchy, there is a need to examine whether such deprivation is associated with T2DM incidence, in addition to the traditional measures of SEP.

We hypothesized that people experiencing material deprivation will have an increased risk on T2DM incidence, in addition to the risk incurred by traditional SEP measures. To test this hypothesis, we used longitudinal data from the Maastricht Study to analyze the influence of adulthood material deprivation independent of other aspects of SEP (education, occupation and income) on T2DM incidence. As this study focused on the impact of material deprivation in adulthood, we controlled for the long-lasting influence of childhood income inadequacy [5]. Childhood income inadequacy is a measurement of childhood poverty. It indicates whether people experienced their family’s income as sufficient for purchasing or replacing necessities such as food and clothes in their childhood. Using an extended measure of material deprivation also allowed the study of the relative importance of its sub-components lack of basic goods, debts, economic strain, and perceived financial problems.

### Methods

We used data from The Maastricht Study, an observational prospective population-based cohort study. The rationale and methodology have been described previously [18]. In brief, the study focuses on the etiology, pathophysiology, complications and comorbidities of T2DM and is characterized by an extensive phenotyping approach. Eligible for participation were all individuals aged between 40 and 75 years and living in the southern part of the Netherlands. Participants were recruited through mass media campaigns and from the municipal registries and the regional Diabetes Patient Registry via mailings. Recruitment was stratified according to known T2DM status, with an oversampling of individuals with T2DM, for reasons of efficiency. The current study includes longitudinal data from 9,187 participants, who completed the baseline survey between November 2010 and October 2020. The examinations of each participant were performed within a time window of three months. The study has been approved by the institutional medical ethical committee (NL31329.068.10) and the Minister of Health, Welfare and Sports of the Netherlands (Permit 131088-105234-PG). All participants gave written informed consent. The participants were followed up to twelve years after baseline (median (IQR) 8.2 (4.9) years). Annual follow-up data were available for 89.2% (year 1), 82.7% (year 2), 79.5% (year 3), 72.4% (year 4), 67.4% (year 5), 58.3% (year 6), 57.9% (year 7), 53.8% (year 8), 43.7% (year 9), 27.5% (year 10) and 12.6% (year 11) of these participants at time of analyses, although follow-up is ongoing. After excluding participants with T2DM at baseline (*n* = 2,058), participants without follow-up data (*n* = 326), and participants with missing responses in the study variables (*n* = 2,250), a total of 4,553 complete cases remained, see Supplementary Figure 1.

### Measures

#### T2DM survival time

At baseline, T2DM was defined according to the WHO 2006 criteria [19]. All participants underwent a standardized oral glucose tolerance test (OGTT) following an overnight fast. Blood samples were collected at baseline and 120 min after ingestion of a 75-gram glucose solution. Participants receiving insulin therapy or those with a fasting glucose level exceeding 11.0 mmol/L (as determined by finger-prick testing) were excluded from the OGTT. Individuals on diabetes medication who had not been previously diagnosed with type 1 diabetes were classified as having T2DM. Incident T2DM was assessed through self-reported annual follow-up questionnaires. Participants were asked: “Has a doctor told you that you have diabetes in the last 12 months?”. As the exact date of the T2DM diagnosis is unknown but has occurred somewhere between the previous and current follow-up questionnaire, the survival time for incident T2DM was calculated as the midpoint between those questionnaires. For censored cases, survival time was defined as the date of the last available follow-up.

#### Material deprivation

The measure for material deprivation was based on the instrument developed by the Netherlands Institute for Social Research [10]. The measure is based on 4 subscales with a total of 20 subitems and was measured at baseline. The first subscale, lack of basic goods, includes 7 items that lacked due to financial reasons: a freezer, refrigerator, car, oven, washing machine, own house and telephone (yes coded as “1” and no coded as “0”). The second subscale, debts, included 3 questions on arrears of payment in mortgage or rent, utility bills and installment payments (yes coded as “1” and no coded as “0”). For the third subscale, economic strain, 6 questions were asked to determine if respondents had enough money for one or more of the following activities: weeklong holiday away from home, meal with meat, chicken or fish every day, adequately heating the home, buying new furniture when needed, buying new clothes when needed, and invite family or friends over for dinner (yes coded as “0” and no coded as “1”). The fourth subscale of perceived financial problems included 4 questions answered on a 5-point Likert scale. The subitems asked about managing the fixed living expenses in the household, the difficulty in paying back debts and the difficulty making ends meet with the monthly income. The answers ranged from “not difficult at all” to “very difficult”. Responses “difficult” and “very difficult” were coded as “1” for deprivation, the remaining answer categories were coded “0”. The fourth subitem asked about the expected change in financial situation for the household in the upcoming 12 months and answer categories ranged from “definitely improve” to “definitely worsen”. Responses “worsen” and “definitely worsen” were coded as “1” for deprivation and the remaining answer categories were coded “0”. To compute an overall indicator for material deprivation, the scores for all subitems (coded “0” or “1”) were cumulated, with a score ranging from 0 to 20. Based on the distribution (see Supplementary Table 1) an overall material deprivation was categorized as none (0 subitems scores as deprived), intermediate deprivation (1 subitem was scored as deprived) and deprived (2 or more subitems scores as deprived).

#### Adult socioeconomic position

This study assessed adult socioeconomic position using three indicators: (i) highest completed level of education, (ii) equivalent household income, and (iii) occupational position. All indicators of SEP were measured at baseline. Participants reported their highest attained education in categories. Education was categorized as none, (un-) completed primary, lower vocational, intermediate vocational, higher general secondary education, higher vocational or university education. For analysis, educational attainment was grouped into tertiles: low (no education, (un)completed primary education, lower vocational education or intermediate general secondary education), intermediate (intermediate vocational education or higher general secondary education), and high (higher vocational or university education). Household income was assessed by asking participants to indicate their net monthly household income within 19 predefined categories (ranging from <€750 to >€5000). The midpoint of each reported household income category (set at €600 for the lowest category and €6000 for the highest category) was divided by the square root of the household size to obtain an equivalent income per person [20, 21]. Occupational position was determined based on responses to an open-ended question regarding current occupation. These responses were coded according to the International Standard Classification of Occupations 2008 (ISCO-08) by a trained coder [22]. The resulting ISCO-08 codes were then converted into the International Socio-Economic Index of Occupational Status (ISEI-08), providing a continuous measure of occupational status. Both income category and occupational position were categorized into tertiles for analysis.

#### Childhood income inadequacy

Childhood income adequacy was assessed with the following question: “Was the financial situation at your childhood’s home sometimes such that there wasn’t enough money to buy food or replace outworn clothes or shoes?”. Responses were recorded on a four-point scale: (1) ‘no, never’, (2) ‘yes, sometimes’, (3) ‘yes, often’ or (4) ‘yes, always’.

#### Covariates

Covariates, measured at baseline, included age, sex and marital status. Marital status was classified into four categories: (1) single, (2) married, in a domestic partnership, civil union or cohabiting, (3) widowed and (4) divorced or separated.

### Statistical analyses

Material deprivation, adult SEP, childhood income inadequacy and demographic characteristics were compared according to T2DM incidence. Adult SEP, childhood income inadequacy and demographic characteristics were also compared across material deprivation groups. To assess the relationships between material deprivation, adult SEP, childhood income inadequacy and incident T2DM, Cox proportional hazards regression analyses were conducted. Models were built up in a stepwise manner. The first model analyzed material deprivation, adult SEP and childhood income inadequacy on T2DM incidence univariately. The second model was adjusted for age, sex, and marital status. The third model was additionally adjusted for all five SEP indicators. The proportional hazard assumption was tested by interaction tests prior to survival analysis; it was not found violated. The four subscales of material deprivation were also analyzed in a stepwise manner in supplementary analyses. Due to the low number of new T2DM cases within these subgroups, the additional analyses for subscales of material deprivation were run with dichotomized measurements (no deprivation versus any deprivation). To check the robustness of the results, measures of material deprivation, adult SEP and childhood income inadequacy were analyzed in the Cox proportional hazard regression analyses as both categorical and continuous variables. Interactions of demographic factors, adult SEP and childhood income inadequacy with material deprivation were tested. The significance level was set at α = 0.05. All analyses were conducted in SPSS 28 [23].

## Results

### Descriptive statistics

Of the original cohort of The Maastricht Study (*n* = 9,187), 4,553 respondents remained in the study sample after exclusion of respondents with T2DM at baseline (*n* = 2,058), without follow-up data (*n* = 326) and with missing responses in the study variables (*n* = 2,250). Of the 4,553 respondents included in this study, 52.4% were female and the mean (SD) age at baseline was 57.9 (8.6) years. The majority of the respondents was married, in a domestic partnership, civil union or living together (80.3%). The number of missing values per variable is reported in Supplementary Table 2. The descriptive statistics for the respondents without T2DM at baseline can be found in Supplementary Table 3. Material deprivation, adult SEP, childhood income inadequacy and demographic characteristics are reported according to T2DM incidence in Table 1. Incident T2DM was 3.2% in the study sample (144 respondents). Incident T2DM was higher for people with intermediate (4.2%) and high material deprivation (5.2%), compared to people who did not experience material deprivation (2.9%, *p* = 0.001). Incident T2DM was also higher for people with low versus high education (5.3% vs. 2.4%, *p* < 0.001), low versus high income (4.4% vs. 1.8%, *p* < 0.001), low versus high occupational position (4.7% vs. 2.5%, *p* = 0.002) and people who experienced income inadequacy in childhood often or all the time versus never (5.9% vs. 2.9%, *p* = 0.046). Furthermore, incident T2DM was more common for people who were older or were male (3.9% vs. 2.5% for females, *p* = 0.009) or widowed (5.3% vs. 1.9% for singles, *p* = 0.167).

**Table 1:** Descriptive statistics (*n* = 4,553)

Variable	*N* (%) or Mean (SD)	*Total*	*T2DM status*		
			No incident T2DM	Incident T2DM	*p*-value
Material deprivation	High deprivation (> = 2 items)	578 (12.7%)	548 (94.8%)	30 (5.2%)	0.001
	Intermediate deprivation (1 item)	723 (15.9%)	693 (95.9%)	30 (4.1%)	
	No deprivation (0 items)	3,252 (71.4%)	3,168 (97.4%)	84 (2.6%)	
Education	Low	1,057 (23.2%)	1,001 (94.7%)	56 (5.3%)	< 0.001
	Intermediate	1,254 (27.5%)	1,219 (97.2%)	35 (2.8%)	
	High	2,242 (49.2%)	2,189 (97.6%)	53 (2.4%)	
Income category	Low	1,532 (33.6%)	1,464 (95.6%)	68 (4.4%)	< 0.001
	Intermediate	1,419 (31.2%)	1,372 (96.7%)	47 (3.3%)	
	High	1,602 (35.2%)	1,573 (98.2%)	29 (1.8%)	
Occupational position	Low	1,148 (25.2%)	1,094 (95.3%)	54 (4.7%)	0.002
	Intermediate	1,643 (36.1%)	1,597 (97.2%)	46 (2.8%)	
	High	1,762 (38.7%)	1,718 (97.5%)	44 (2.5%)	
Childhood income inadequacy	Often, all the time	186 (4.1%)	175 (94.1%)	11 (5.9%)	0.046
	Sometimes	752 (16.5%)	724 (96.3%)	28 (3.7%)	
	Never	3,615 (79.4%)	3,510 (97.1%)	105 (2.9%)	
Age		57.9 (8.6)	57.9 (8.6)	59.7 (8.2)	0.010
Sex	Female	2,386 (52.4%)	2,326 (97.5%)	60 (2.5%)	0.009
	Male	2,167 (47.6%)	2,083 (96.1%)	84 (3.9%)	
Marital status	Single	378 (8.3%)	371 (98.1%)	7 (1.9%)	0.167
	Married, domestic partnership, civil union, or cohabiting	3,657 (80.3%)	3,537 (96.7%)	120 (3.3%)	
	Widowed	152 (3.3%)	144 (94.7%)	8 (5.3%)	
	Divorced, separated	366 (8.0%)	357 (97.5%)	9 (2.5%)	

*SD* standard deviation, *T2DM* type 2 diabetes mellitus

Supplementary Table 4 provides the distribution of number of subitems scored for each of the four components of material deprivation. Perceived financial problems were most common with 22.0% of respondents answering at least once that they experienced problems in that area, followed by 12.8% of respondents indicating economic strain, 6.4% of respondents reporting a lack of basic goods due to financial problems and 1.4% of respondents reporting debts.

Table 2 reports adult SEP, childhood income inadequacy and demographic characteristics for people across material deprivation groups (none, intermediate or high). High material deprivation was more common for people with low versus high education (20.2% vs. 8.7%, *p* < 0.001), low versus high income (28.5% vs. 2.0%, *p* < 0.001) and low versus high occupational position (21.6% vs. 8.5%, *p* < 0.001) and people who experienced income inadequacy in childhood often or all the time versus never (26.3% vs. 10.7%, *p* < 0.001). Material deprivation was also more common for females (15.4%) compared to males (9.7%, *p* < 0.001), and more common for single (25.9%), widowed (15.1%) or divorced people (36.1%) compared to married people (8.9%, *p* < 0.001).

**Table 2:** Adult SEP, childhood income inadequacy and demographic factors per material deprivation group (*n* = 4,553)

	*N* (%) or Mean (SD)	Total	Material deprivation group			*p*-value
			None	Intermediate	High	
Highest level of completed education	Low	1,057 (23.2%)	669 (63.3%)	174 (16.5%)	214 (20.2%)	< 0.001
	Intermediate	1,254 (27.5%)	876 (69.9%)	208 (16.6%)	170 (13.6%)	
	High	2,242 (49.2%)	1,707 (76.1%)	341 (15.2%)	194 (8.7%)	
Household income	Low	1,532 (33.6%)	807 (52.7%)	288 (18.8%)	437 (28.5%)	< 0.001
	Intermediate	1,419 (31.2%)	1,102 (77.7%)	208 (14.7%)	109 (7.7%)	
	High	1,602 (35.2%)	1,343 (83.8%)	227 (14.2%)	32 (2.0%)	
Occupational position	Low	1,148 (25.2%)	708 (61.7%)	192 (16.7%)	248 (21.6%)	< 0.001
	Intermediate	1,643 (36.1%)	1,203 (73.2%)	259 (15.8%)	181 (11.0%)	
	High	1,762 (38.7%)	1,341 (76.1%)	272 (15.4%)	149 (8.5%)	
Childhood income inadequacy	Often, all the time	186 (4.1%)	94 (50.5%)	43 (23.1%)	49 (26.3%)	< 0.001
	Sometimes	752 (16.5%)	465 (61.8%)	144 (19.1%)	143 (19.0%)	
	Never	3,615 (79.4%)	2,693 (74.5%)	536 (14.8%)	386 (10.7%)	
Age		57.9 (8.6)	57.9 (8.6)	58.8 (8.6)	57.1 (8.5)	0.002
Sex	Female	2,386 (52.4%)	1,643 (68.9%)	375 (15.7%)	368 (15.4%)	< 0.001
	Male	2,167 (47.6%)	1,609 (74.3%)	348 (16.1%)	210 (9.7%)	
Marital status	Single	378 (8.3%)	232 (61.4%)	48 (12.7%)	98 (25.9%)	< 0.001
	Married, domestic partnership, civil union, or living together	3,657 (80.3%)	2,754 (75.3%)	578 (15.8%)	325 (8.9%)	
	Widowed	152 (3.3%)	96 (63.2%)	33 (21.7%)	23 (15.1%)	
	Divorced, separated	366 (8.0%)	170 (46.4%)	64 (17.5%)	132 (36.1%)	

*SEP *socioeconomic position, *SD *standard deviation

### Longitudinal associations

Table 3 shows the longitudinal associations of material deprivation, adult SEP and childhood income inadequacy with incident T2DM. In models 1 (crude model) and 2 (adjusted for demographic factors), all five factors were associated with higher risk for T2DM, in the expected direction. Independent of sex, age and marital status, people who faced material deprivation had a higher risk for T2DM with a hazard ratio (HR) of 2.41 (confidence interval (CI), 1.57 to 3.70). Similar higher HRs for T2DM were found for people with low education (2.25 CI (1.53–3.31)), low income (2.69 CI (1.73–4.17)), low occupation (2.06 CI (1.37–3.07)) and people who reported childhood income inadequacy often or always (2.09 CI (1.12–3.90)). In the third model of Table 3, which includes demographic factors and all SEP indicators, two significant associations remain. People with high material deprivation and people with intermediate or low income had higher risks for incident T2DM, independent of demographic factors, level of education, occupational status and childhood income inadequacy. The HR of material deprivation was 1.71 (CI 1.08–2.70). Interactions were checked for material deprivation with age, sex, marital status and the other SEP indicators for T2DM incidence; none were significant.

**Table 3:** Hazard ratios T2DM for material deprivation, education, income, occupational position and childhood income inadequacy, results from Cox regression analyses (*n* = 4,553)

	HR (95% CI)	Model 1	Model 2	Model 3
Material deprivation	None	ref	ref	Ref
	Intermediate	**1.54 (1.01–2.33)**	**1.52 (1.00-2.31)**	1.38 (0.90–2.10)
	High	**2.05 (1.35–3.11)**	**2.41 (1.57–3.70)**	**1.71 (1.08–2.70)**
Educational level	High	ref	ref	ref
	Intermediate	1.16 (0.76–1.77)	1.21 (0.79–1.86)	0.94 (0.58–1.53)
	Low	**2.28 (1.57–3.32)**	**2.25 (1.53–3.31)**	1.46 (0.88–2.44)
Occupational position	High	ref	ref	Ref
	Intermediate	1.13 (0.75–1.70)	1.20 (0.79–1.82)	0.98 (0.62–1.55)
	Low	**1.97 (1.32–2.94)**	**2.06 (1.37–3.07)**	1.22 (0.72–2.07)
Income level	High	ref	ref	ref
	Intermediate	**1.80 (1.13–2.86)**	**1.95 (1.23–3.11)**	**1.74 (1.08–2.80)**
	Low	**2.44 (1.58–3.77)**	**2.69 (1.73–4.17)**	**1.86 (1.12–3.08)**
Childhood income inadequacy	Never	ref	ref	ref
	Sometimes	1.27 (0.84–1.93)	1.23 (0.81–1.87)	1.05 (0.69–1.60)
	Often, always	**2.09(1.12–3.90)**	**2.09 (1.12–3.90)**	1.67 (0.89–3.14)

Significant results in bold

Model 1 is the crude model of a single SEP indicator

Model 2 adjusts the crude model for sex, age and marital status

Model 3 adjusts for sex, age, marital status, and for all 5 SEP indicators mutually

*T2DM* type 2 diabetes mellitus, *HR* hazard ratio, *CI* confidence interval

To check the robustness of the results for material deprivation and T2DM incidence, Supplementary Table 5 reports the results for the continuous measurement of the variables. Models 1 and 2 show similar results for all 5 SEP indicators. After adjustment for demographic factors and mutual adjustments for all 5 SEP indicators in one model, material deprivation and education remain significant. The HR for material deprivation is 1.26 (CI 1.10–1.44).

As material deprivation was comprised of four subscales, it was possible to conduct more detailed analyses for the different components of material deprivation (lack of goods due to financial reasons, debts, economic strain and perceived financial problems). Supplementary Table 6 reports associations for all 4 subscales and T2DM incidence in the expected direction, especially in models 1 and 2. Overall, having debts showed the strongest association with HRs of 2.65 (CI 1.09–6.48) in the crude model, and 2.27 (CI 0.87–5.94) in the fully adjusted model (including all demographic and SEP factors). Next, lack of basic goods due to financial reasons was associated with higher risk for T2DM with HR 1.34 (CI 0.71–2.52) and perceived financial problems with an HR of 1.33 (CI 0.90–1.96) in the fully adjusted model. Economic strain showed the lowest (elevated) risk for T2DM with a HR of 1.09 (CI 0.66–1.82) in the fully adjusted model.

## Discussion

In this research, we aimed to analyze the influence of material deprivation independent of education, income, occupation and childhood income inadequacy on T2DM incidence in a large cohort study with up to 12 years of follow-up. The risk for incident T2DM was higher for people who face high material deprivation, independent of their age, sex, marital status, level of highest completed education, income and occupational level and childhood income inadequacy. The results therefore suggest an association between material deprivation and T2DM incidence, independent of traditional SEP measures.

The results of this study show the longitudinal associations of material deprivation on T2DM. They extend previously established associations between material deprivation and T2DM prevalence in a cross-sectional study [3]. Together, these studies show that material deprivation increases the risk of T2DM prevalence and incidence, in addition to traditional determinants of SEP. The findings of this study also suggest that material deprivation in adulthood is associated with T2DM incidence, independently of the long-lasting influence of childhood income inadequacy [5]. The results indicate that individual-level material deprivation may represent another risk factor for T2DM, complementing previous studies that have identified adverse health associations of material deprivation at the area level [14–17]. Material deprivation is a more multidimensional construct that encompasses the inability to meet basic needs, access to healthcare or social participation [3, 8, 10]. Both low income and material deprivation are associated with behavioral risk factors for T2DM and stress [7]. The results seem to support that material deprivation may imply more acute stress and more acute, tangible constraints on health-related behaviors. Furthermore, material deprivation seems to better capture cumulative disadvantage across the life- course. This may be particularly relevant for T2DM, a condition influenced by both early-life and adulthood socioeconomic conditions [2–5]. Finally, material deprivation may account for structural and environmental determinants, such as residential and food insecurity, more appropriately than income alone.

Different policy definitions for poverty exist in the Netherlands. To improve clarity and consistency in poverty definitions, Statistics Netherlands, the National Institute for Family Finance Information (Nibud) and the Netherlands Institute for Social Research (SCP) joined forces in defining poverty for the period of 2018–2023. These institutes adjusted household income for household size, the number of parents and children, the age of children, living expenses and the presence of a financial buffer. Living expenses include rent, utility bills, insurances, clothing, groceries and costs for social participation (subscriptions for library, sport, music, hobby or cultural associations and costs for telephone and internet access). The financial buffer is defined as at least one year’s worth of income at the poverty standard for that type of household. If a sufficient financial buffer is present, the household is defined as not living in poverty. The report established 35 different types of households and their necessary budget to fully participate in society, reflecting the concept of *relative poverty*. In other words, poverty is not conceptualized as merely an absolute lack of resources. Using this new measure, the report shows that 540.000 people in the Netherlands were considered poor in 2023 (3.1% of the population) and another 1.2 million (6.9%) had an income near the poverty standard (up to 125%) with a limited financial buffer [24]. With 10% of the people living in or near poverty [24], the wealthy Netherlands is not wealthy for everybody.

The region where the current study took place is also a more impoverished region (the South of Limburg [25, 26]), with 4 of its cities (including Maastricht) ranking in the national top 15 of cities with the highest percentage of people living in poverty [27]. In our study, poverty was not only defined as having an income below a certain threshold, but the concept of material deprivation was used in addition to income level. Furthermore, the concept is multifaceted and the measurement that we used allows for further specification of people who face deprivation. In fact, we show in our study sample that 15.9% faced deprivation on at least one subitem, and another 12.7% faced deprivation on two or more subitems of the material deprivation scale. Given the prevalence of poverty in the study area and given the current findings on its toxic effects on a highly prevalent chronic condition, we emphasize the need to strengthen measures that aim to tackle poverty, particularly in said region.

In addition to strengthening measures in practice, we also suggest future research to include broader material deprivation measurements in determination of SEP. This aligns with a broader international perspective moving beyond purely income-based thresholds to multidimensional concepts of poverty reflecting relative disadvantage in a social context. For example, the European Union defines individuals as at risk of poverty or social exclusion if the household income is below 60% of the national median, or if households face severe material and social deprivation or low work intensity. In 2024, 93.3 million people in the EU were at risk of poverty or social exclusion (21.0% of the total population) [28].

Several strengths of this study are related to its design. First, the study allowed for follow-up of 4,553 respondents for a period up to 12 years, with a median follow-up of 8.2 years. This enabled longitudinal analyses which help to better analyze the influence of different measures of SEP in adulthood and childhood on T2DM incidence. This research could therefore build upon previously established cross- sectional associations between material deprivation and prevalent T2DM [3] and further explore the potential relationship between material deprivation and increased risk for incident T2DM. Second, this study used a list of material deprivation items and subcomponents. This allowed for more in-depth analysis between different subcomponents of material deprivation, such as having debts, lack of basic goods due to financial reasons, perceived financial problems and economic strain. Third, extensive data on SEP measures enabled stringent control for the traditional measures of SEP, simultaneously. Fourth, data on the recalled childhood income inadequacy enabled the examination of associations between current deprivation in middle and older age and T2DM, after accounting for the previously reported long-lasting influence of childhood deprivation previously reported [5] on T2DM risk.

Limitations of this study should also be noted. First, the stringent control for traditional measures of SEP might have introduced multicollinearity. Multicollinearity might become more problematic with categorical than with continuous measures. This might underlie why income remains statistically significant in the categorical analyses and education remains statistically significant in the continuous analyses. Second, relatively healthy and high SEP respondents may be more likely to have participated in the study sample [29]. Respondents with low SEP and/or less healthy respondents were more likely to drop out of the study compared to respondents with high SEP and/or more healthy respondents [30]. According to the healthy survivor bias, it cannot be excluded that participants with low SEP and/or poorer health are less likely to continue follow-up due to shorter life expectancies. A comparison between the original sample of people without T2DM at baseline (*n* = 7,129) and the final included sample (*n* = 4,553), showed that indeed low SEP respondents were more likely to drop out of the study during the study period (Supplementary Table 3). These sample patterns would make the results of this study conservative, or underestimations of the true effects of material deprivation and incident T2DM. Third, it cannot be excluded that the accuracy of using self-reporting new cases of T2DM, compared to using a standardized oral glucose tolerance test, may vary between socioeconomic groups [31]. This would also make the results of this study conservative, or underestimations. Fourth, the current study does not include area-level indicators of SEP [32]. In previous studies, reported Intraclass Correlation Coefficients are small which indicates that differences within neighborhoods are greater than the differences between neighborhoods. The current study also did not include variables indicating lifestyle and biological pathways that might be in the causal pathway and this would risk overadjustment of the studied associations. Fifth, only 144 incident T2DM cases occurred among the 4,553 participants, providing limited power. Consequently, more subtle associations cannot be detected. Larger cohorts with more events are needed to confirm these findings. Finally, over 95% of the sample was of Western-European descent as the study was based on a local population (the city of Maastricht and its surroundings). This limitation did not allow for analyses of people with different ethnic backgrounds.

## Conclusion

Higher levels of material deprivation are associated with an increased risk of T2DM, even after accounting for other socioeconomic factors. It is important to use extended measures of socioeconomic circumstances to better take account of differences between people at the very bottom of the socioeconomic hierarchy. Even in the relatively wealthy Netherlands, material deprivation is not uncommon and may increase the risk of T2DM incidence. This warrants more attention in research and practice for people with material deprivation, in addition to well-known SEP indicators such as income, education and occupation. Interventions aimed at reducing health inequalities should also target multiple SEP components and pathways.

## Supplementary Information

Supplementary Material 1.

## Data Availability

The datasets used and/or analyzed during the current study are not publicly available due to privacy of individuals that participated in the study. The data are available from the corresponding author on reasonable request.
