# Supplementary material for: The importance of material deprivation in type 2 diabetes incidence: longitudinal analyses in the Maastricht study
Source: BMC Public Health. 2025 Sep 2;25:3013. doi: 10.1186/s12889-025-24263-1 (PMC12403942; doi:10.1186/s12889-025-24263-1)
Supplement: Supplementary file 1 — Supplementary Material 1. [file 12889_2025_24263_MOESM1_ESM.docx]

**Supplementary Figure 1. Flowchart complete case sample**

9,187 respondents

6,803 respondents without diabetes at baseline and follow-up data

7,129 respondents without diabetes at baseline

2,058 respondents with diabetes at baseline

326 respondents no follow-up data available

34 marital status

76 education,
1,469 income,
739 occupation,
327 childhood income
inadequacy

2,250 respondents with missing responses in study variables.

355 lack of basic goods for financial reasons,
340 debts,
582 economic strain,
488 perceived financial problems

4,553 complete cases

| **Supplementary Table 1. Distribution of material deprivation, number of subitems scored as deprived (n=4,553)** | | | |  |  |
| --- | --- | --- | --- | --- | --- |
|  | N | % | |  |  |
| 0 | 3,252 | 71.4 | |  |  |
| 1 | 723 | 15.9 | |  |  |
| 2 | 169 | 3.7 | |  |  |
| 3 | 121 | 2.7 | |  |  |
| 4 | 76 | 1.7 | |  |  |
| 5 | 67 | 1.5 | |  |  |
| 6 | 57 | 1.3 | |  |  |
| 7 | 27 | 0.6 | |  |  |
| 8 | 30 | 0.7 | |  |  |
| 9 | 12 | 0.3 | |  |  |
| 10 | 9 | 0.2 | |  |  |
| 11 | 6 | 0.1 | |  |  |
| 12 | 2 | 0 | |  |  |
| 13 | 2 | 0 | |  |  |
| **Supplementary Table 2. Missing values (n=6,803)** | | |  | |  |
|  | | | N | | % |
| Age | | | 0 | | 0.0 |
| Sex | | | 0 | | 0.0  0 |
| Marital status | | | 34 | | 0.5 |
| Education | | | 76 | | 1.1 |
| Income category | | | 1,469 | | 21.6% |
| Occupational status | | | 739 | | 10.9% |
| Childhood income inadequacy | | | 327 | | 4.8% |
| Missed paying rent/mortgage past year | | | 329 | | 4.8% |
| Missed paying utility bills past year | | | 333 | | 4.9% |
| Missed an installment payment past year | | | 337 | | 5.0% |
| Enough money to go on holiday | | | 399 | | 5.9% |
| Enough money to heat your home | | | 343 | | 5.0% |
| Enough money to change worn out furniture | | | 457 | | 6.7% |
| Enough money to buy new clothes | | | 406 | | 6.0% |
| Enough money to invite others for dinner | | | 387 | | 5.7% |
| Enough money to for warm meal with meat | | | 332 | | 4.9% |
| Difficulty paying fixed monthly expenses | | | 429 | | 6.3% |
| Difficulty making ends meet with monthly income | | | 326 | | 4.8% |
| Difficulty paying back debts | | | 397 | | 5.8% |
| Expected change of financial situation | | | 328 | | 4.8% |
| Financial reason no freezer | | | 332 | | 4.9% |
| Financial reason no car | | | 331 | | 4.9% |
| Financial reason no oven | | | 335 | | 4.9% |
| Financial reason no washing machine | | | 330 | | 4.9% |
| Financial reason no house | | | 333 | | 4.9% |
| Financial reason no fridge | | | 331 | | 4.9% |
| Financial reason no phone | | | 331 | | 4.9% |

| **Supplementary Table 3. Descriptive statistics for respondents without T2DM at baseline** | | |
| --- | --- | --- |
| Variable |  | N (%) or Mean (SD) |
| Education (n=7,049) | Low | 2,068 (29.3%) |
|  | Intermediate | 1,958 (27.8%) |
|  | High | 3,023 (42.4%) |
| Income category (n=5,522) | Low | 2,030 (36.8%) |
|  | Intermediate | 1,666 (23.4%) |
|  | High | 1,826 (25.6%) |
| Occupational position (n=6,338) | Low | 1,851(29.2%) |
|  | Intermediate | 2,259 (35.6%) |
|  | High | 2,228 (35.2%) |
| Childhood income inadequacy (n=6,723) | Often, all the time | 295 (4.4%) |
|  | Sometimes | 1,152 (17.1%) |
|  | Never | 5276 (78.5%) |
| Age |  | 58.6 (8.8) |
| Sex (n=7,129) | Female | 3,918 (55.0%) |
|  | Male | 3,211 (45.0%) |
| Marital status (n=7,093) | Single | 581 (8.1%) |
|  | Married, domestic partnership, civil union, or cohabiting | 5611 (79.1%) |
|  | Widowed | 273 (3.8%) |
|  | Divorced, separated | 600 (8.4%) |

T2DM: type 2 diabetes mellitus. SD: standard deviation.

| **Supplementary Table 4. Distribution of subscales of material deprivation, number of subitems scored as deprived (n=4,553)** | | |
| --- | --- | --- |
|  | N=0 (%) | N>=1 (%) |
| Lack of goods due to financial reasons | 4,261 (93.6%) | 292 (6.4%) |
| Debts | 4,487 (98.6%) | 66 (1.4%) |
| Economic strain | 3,968 (87.2%) | 585 (12.8%) |
| Perceived financial problems | 3,551 (78.0%) | 1,002 (22.0%) |

| **Supplementary Table 5. Hazard ratios T2DM for continuous SEP indicators, results from Cox regression analyses (n=4,553).** | | | |
| --- | --- | --- | --- |
| HR (95% CI) | Model 1 | Model 2 | Model 3 |
| Material deprivation | **1.30 (1.16-1.45)** | **1.41 (1.25-1.59)** | **1.26 (1.10-1.44)** |
| Highest level of completed education | **1.52 (1.30-1.78)** | **1.49 (1.27-1.75)** | **1.28 (1.03-1.59)** |
| Occupational position | **1.34 (1.14-1.58)** | **1.38 (1.17-1.62)** | 1.02 (0.82-1.27) |
| Household income | **1.46 (1.21-1.78)** | **1.57 (1.28-1.92)** | 1.21 (0.96-1.52) |
| Childhood income inadequacy | **1.17 (1.02-1.34)** | **1.17 (1.02-1.34)** | 1.06 (0.92-1.23) |

T2DM: type 2 diabetes. SEP: socioeconomic position. HR: hazard ratio. CI: confidence interval. Model 1 is the crude model of a single SEP indicator. Model 2 adjusts the crude model for sex, age and marital status. Model 3 adjusts for sex, age, marital status, and all 5 SEP indicators. Significant results in bold.

| **Supplementary Table 6. Hazard ratios T2DM for material deprivation, results from Cox regression analyses (n=4,553).** | | | | | |
| --- | --- | --- | --- | --- | --- |
|  | HR (95% CI) | Model 1 | Model 2 | Model 3 |  |
| Lack of basic goods due to financial reasons | Not deprived | ref | ref | ref |  |
|  | Deprived | **1.83 (1.07-3.13)** | **2.42 (1.36-4.31)** | 1.34 (0.71-2.52) |  |
| Debts | Not deprived | ref | ref | Ref |  |
|  | Deprived | **2.65 (1.09-6.48)** | **3.51 (1.41-8.78)** | 2.27 (0.87-5.94) |  |
| Economic strain | Not deprived | ref | ref | ref |  |
|  | Deprived | **1.73 (1.15-2.62)** | **1.96 (1.28-2.99)** | 1.09 (0.66-1.82) |  |
| Perceived financial problems | Not deprived | ref | ref | ref |  |
|  | Deprived | **1.64 (1.16-2.32)** | **1.69 (1.19-2.40)** | 1.33 (0.90-1.96) |  |

T2DM: type 2 diabetes. HR: hazard ratio. CI: confidence interval. Respondents who scored 0 on all subitems were considered not deprived, respondents scoring 1 subitem or more were considered deprived. Model 1 is the crude model of a single SEP indicator. Model 2 adjusts the crude model for sex, age and marital status. Model 3 adjusts for sex, age, marital status, and all 5 SEP indicators.
